# *Microbacterium elymi* sp. nov., Isolated from the Rhizospheric Soil of *Elymus tsukushiensis*, a Plant Native to the Dokdo Islands, Republic of Korea

**DOI:** 10.4014/jmb.2211.11024

**Published:** 2023-01-06

**Authors:** Ye-Ji Hwang, Soo-Yeong Lee, Jin-Soo Son, Jin-suk Youn, Woong Lee, Jae-Ho Shin, Mi-Hwa Lee, Sa-Youl Ghim

**Affiliations:** 1 Microbiology Research Department, Nakdonggang National Institute of Biological Resources, Sangju 37242, Republic of Korea; 2 Molecular Phytobacteriology Laboratory, Infectious Disease Research Center, KRIBB, Daejeon 34141, Republic of Korea; 3 School of Life Sciences, Research Institute for Dok-do & Ulleung-do Island, Kyungpook National University, Daegu 41566, Republic of Korea; 4 School of Applied Biosciences, College of Agriculture and Life Sciences, Kyungpook National University, Daegu 41566, Republic of Korea

**Keywords:** *Microbacterium*, novel bacterium, *Elymus tsukushiensis*, Dokdo Islands

## Abstract

*Microbacterium elymi* KUDC0405^T^ was isolated from the rhizosphere of *Elymus tsukushiensis* from the Dokdo Islands. The KUDC0405^T^ strain was Gram-stain-positive, non-spore forming, non-motile, and facultatively anaerobic bacteria. Strain KUDC0405^T^ was a rod-shaped bacterium with size dimensions of 0.3–0.4 × 0.7–0.8 μm. Based on 16S rRNA gene sequences, KUDC0405^T^ was most closely related to *Microbacterium bovistercoris* NEAU-LLE^T^ (97.8%) and *Microbacterium pseudoresistens* CC-5209^T^ (97.6%). The dDDH (digital DNA-DNA hybridization) values between KUDC0405^T^ and *M. bovistercoris* NEAU-LLE^T^ and *M. pseudoresistens* CC-5209^T^ were below 17.3% and 17.5%, respectively. The ANI (average nucleotide identity) values among strains KUDC0405^T^, *M. bovistercoris* NEAU-LLE^T^, and *M. pseudoresistens* CC-5209^T^ were 86.6% and 80.7%, respectively. The AAI (average amino acid identity) values were 64.66% and 64.97%, respectively, between KUDC0405^T^ and its closest related type strains. The genome contained 3,596 CDCs, three rRNAs, 46 tRNAs, and three non-coding RNAs (ncRNAs). The genomic DNA GC content was 70.4%. The polar lipids included diphosphatydilglycerol, glycolipid, phosphatydilglycerol, and unknown phospholipid, and the major fatty acids were anteiso-C_17:0_ and iso-C_16:0_. Strain KUDC0405^T^ contained MK-12 as the major menaquinone. Based on genotypic, phylogenetic, and phenotypic properties, strain KUDC0405^T^ should be considered a novel species within the genus *Microbacterium*, for which we propose the name *M. elymi* sp. nov., and the type strain as KUDC0405^T^ (=KCTC 49411^T^, =CGMCC1.18472^T^).

## Introduction

The genus *Microbacterium* was classified by Orla-Jensen S [1] based on the d-lactic acid-producing bacteria in milk. At that time, the genus *Microbacterium* comprised 157 species, including *Microbacterium aerolatum* V-73^T^ [2], *M. agarici* CC-SBCK-209^T^ [3], *M. album* SYSU D8007^T^ [4], *M. algeriense* G1^T^ [5], *M. amylolyticum* N5^T^ [6], *M. aoyamense* KV-492^T^ [7], *M. aquimaris* JS54-2^T^ [8]. *Microbacterium* can be isolated from various sources, such as seawater, desert soil, maize rhizosphere, cow dung, and microfiltered milk. Members of this genus are Gram-stain-positive, rod-shaped, and have an optimum growth temperature of 20–30°C. Here, we report a taxonomic analysis of the novel bacterial strain, KUDC0405^T^, isolated from the rhizospheric soil of *Elymus tsukushiensis*, a plant native to the Dokdo Islands (37°14′24.2′′ N, 131°52′12.2′′ E). During microbial diversity monitoring in April 2014, rhizospheric soil samples were collected from native plants of the Ulleungdo and Dokdo Islands (Ulleung-gun, Gyeongsangbuk-do). *E. tsukushiensis* var. *trasiens* (Hack.) Osada is native to the Dokdo Islands and is the dominant plant species on these islands, and its distribution is expanding [9]. The Dokdo Islands are a group of volcanic islands located in the middle of the East Sea, east of the Republic of Korea (37°14′24.2′′N, 131°52′12.2′′E; Uljin-gun, Gyeongsangbuk-do). The island group consists of two large islands (Dong-do and Seo-do) and 89 small islets, and high concentrations of Ba, K, and Rb have been detected in soil [10]. Despite the difficult survival environment for plants, various plants inhabit Dokdo, and various useful microorganisms and novel species of bacteria have been discovered associated with plants and characterized for plant growth-promoting traits. Some of these microorganisms include *Brevibacterium iodinum* KUDC1716 [11], *Ochrobactrum lupini* KUDC1013 and *Novosphingobium pentaromativorans* KUDC1065 [12].

## Materials and Methods

### Microorganism Isolation and Cultivation

Plant samples were collected and stored as described previously [13]. The samples were suspended in 0.85%NaCl (w/v) and serial dilutions (10^-4^‒10^-6^) were prepared. A 100 μL aliquot dilution was plated onto R2A and 1/10 diluted tryptic soy agar (TSA) (Difco, USA) and incubated at 25°C for 7 days. Morphologically different colonies were selected, and individual colonies were further purified by repeated streaking onto TSA media. The type strains used in this study were obtained from the China General Microbiological Culture Collection Centre (CGMCC) and the Korea Collection for Type Cultures (KCTC). The strains were cultivated on TSA at 25°C and maintained at -70°C in saline solution (0.85% NaCl, w/v) supplemented with 15% glycerol (v/v).

### 16S rRNA Gene Sequencing and Phylogenetic Analysis

The phylogenetics of the isolated strain was determined based on a comparative analysis of the 16S rRNA gene sequence. The 16S rRNA gene sequence was amplified and the PCR products were purified as described previously [14]. Universal primer pairs 518F (5′-CCA GCA GCC GCG GTA ATA CG-3′) and 800R (5′-TAC CAG GGT ATC TAA TCC-3′) were used for amplification [15]. Direct sequencing of the 16S rRNA gene was conducted by Macrogen (http://macrogen.com/) using the sequencing primers (518F and 800R) and an automated sequencer (ABI 3730xl; Applied Biosystems, USA). The EzBioCloud server (https://ezbiocloud.net/) [16] was used to identify the closest phylogenetic neighbors and calculate pairwise 16S rRNA gene sequence similarity values with respect to the novel strain.

All 16S rRNA gene sequences of the closest type strains were aligned using CLUSTAL_W [17] and the sequence data were analyzed using BioEdit [18]. Gaps at the 5′ and 3′ ends of the alignment were omitted from further analysis. Bayesian inference was performed using a Markov Chain Monte Carlo (MCMC) algorithm in MrBayes v.3.2.7a [19]. The six simultaneous Markov chains were run for 1,000,000 generations. Trees were started from random trees and saved every 1,000th generation. Burn-in was set at 25% generations. The Jukes and Cantor [20] model was used to generate an evolutionary distance matrix for the neighbor-joining algorithm. Phylogenetic trees were constructed using the neighbor-joining algorithm in PHYLIP version 3.696 [21]. The SEQBOOT and CONSENSE programs in the PHYLIP package were used to evaluate the resulting tree topologies by bootstrap analysis with 1,000 replications [22]. The phylogenetic trees were indicated using maximum parsimony and maximum likelihood [22] tree-making algorithms in the MEGA-X 10.1.8 software [23] with 1,000 bootstrap replicates. Maximum likelihood phylogeny was generated using the Kimura-2-parameter model [24]. *Rathayibacter rathayi* VKM Ac-1601^T^, which is not affiliated with the genus *Microbacterium* was used as an outgroup. Trees were rooted and constructed using MEGA-X in the Newick format.

### Whole-Genome Sequence Analysis

Chromosomal DNA was extracted in accordance with Sambrook *et al*. [25] and was purified as described by Yoon *et al*. [26]. The cell biomass for DNA was cultured on TSA at 30°C for 2 days. The complete genome of the strain KUDC0405^T^ was sequenced using a MinION platform (Oxford Nanopore Technologies, UK). The reads were assembled de novo using Flye (version 2.9) [27]. The automatic NCBI Prokaryotic Genomes Annotation Pipeline (PGAP) [28] and the Rapid Annotation of microbial genomes using the Subsystems Technology (RAST) server [29] were used for combine the complete genome sequence. To identify biosynthetic gene clusters (BGCs) for secondary metabolites using The AntiSMASH server (version 6.0) [30]. The average nucleotide identity (ANI) values based on the blast+ algorithm (ANIb) and the MUMmer (ANIm) ultra-rapid alignment tool between strain KUDC0405^T^ and closely related strains were calculated using the JSpeciesWS website (https://jspecies.ribohost.com/jspeciesws/) [31]. The average amino acid identity (AAI) values were obtained using Kostas lab (http://enve-omics.ce.gatech.edu/) [32]. The dDDH values were calculated using server-based genome-to-genome distance calculator version 2.1 (http://ggdc.dsmz.de/distcalc2.php) [33]. The dDDH results were based on recommended formula 2 (identities/HSP length). Orthologous genes were identified between strain KUDC0405^T^ and its two closest relatives, *M. bovistercoris* NEAU-LLE^T^ and *M. pseudoresistence* CC-5209^T^, using protein sequences annotated by Hyatt *et al*. [33] and the OrthoVenn diagram [34]. A multi-locus species tree based on the whole genome sequences of each reference strain was established using autoMLST (http://automlst.ziemertlab.com) [35].

### Morphological Characteristics

The scanning electron micrograph of strain KUDC0405^T^ was observed with a Zentech digital camera (Sw 804255; Samwon Optics and Seige, Korea) for cell morphology and size, using cells grown on TSA. The cells were treated with 1% osmium tetroxide 25°C for 1 h, and dehydrated with graded series of ethanol (50%, 70%, 80%, 90%, and 100%), followed by isoamyl acetate. After lyophilization, the samples were coated with platinum (20 mA, 90 s), and the cell morphology was observed using a field emission scanning electron microscope (FE-SEM, SU8220; Hitachi, Japan).

### Growth Capabilities and Motility

Growth capability of strain KUDC0405^T^ and the reference strains (*M. bovistercoris* NEAU-LLE^T^ and *M. pseudoresistence* CC-5209^T^) at different temperatures (4, 10, 25, 30, 37, and 42°C) and different pH values (pH 3–12, in 1 pH unit intervals). The pH values were adjusted as described by Kämpfer *et al*. [36] in sterilized TSA. The growth capability in the different NaCl concentrations (0‒10% w/v, in 0.5% increments) was also evaluated in tryptic soy broth (TSB). Oxidase activity was determined using BBL Oxidase Reagent (Becton Dickinson Biosciences, USA), and catalase activity was evaluated in 3% (v/v) hydrogen peroxide solution.

### Physiological and Chemotaxonomical Characteristics

To determine hydrolysis of starch, urea, Tween 20, 40, 60, and 80, the isolate was cultured on TSA at 30°C for a week, as described by Cowan and Steel [37]. The enzyme activity was evaluated using API ZYM kit, API 20NE kit (bioMérieux, France), and acid production was tested API 50CH test (bioMérieux, France) according to the manufacturer’s instructions at 30°C for 2 days. The type strains, *M. bovistercoris* NEAU-LLE^T^ and *M. pseudoresistence* CC-5209^T^, which are related to KUDC0405^T^, were analyzed under the same conditions. The cell wall peptidoglycan was analyzed using an amino acid analyzer (L-8900; Hitachi, Japan). To analyze the polar lipids, two-dimensional thin layer chromatography (TLC) analysis were used according to Minnikin *et al*. [38]. The fatty acid composition analysis was performed using the Microbial Identification System from cells of the strain KUDC0405^T^, and reference strains were incubated on TSA at 30°C for 7 days. To determine siderophore production by strain KUDC0405^T^, chrome azurol S (CAS) media were used as previously described [39, 40].

## Results and Discussion

### Identification by 16S rRNA Gene Sequencing and Phylogenetic Analysis

**Fig. 1:**
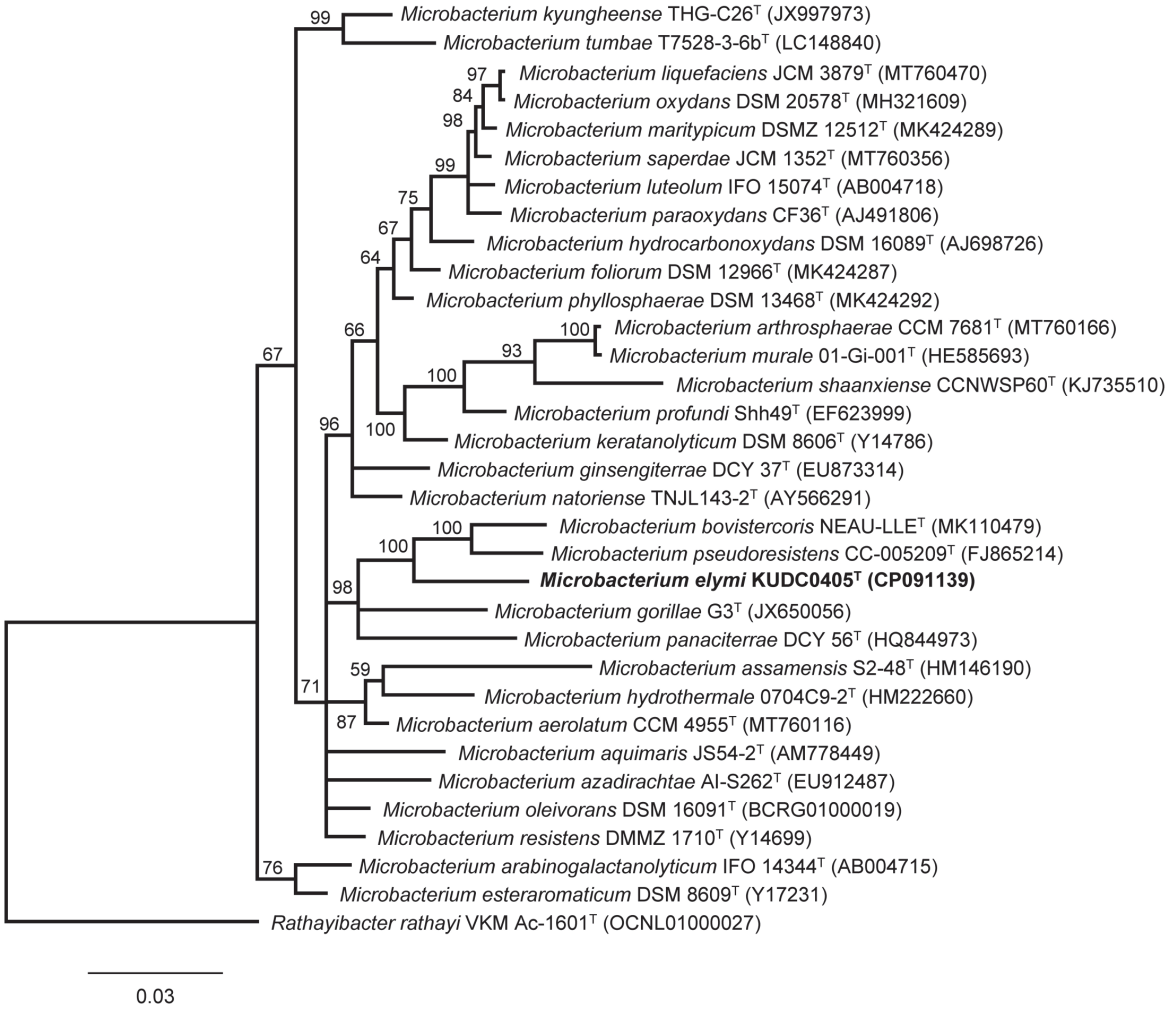
Phylogenetic tree using the Bayesian inference method indicating the relationships between stain KUDC0405^T^ and related type strains. Bayesian inference tree, reconstructed by comparative analysis of 16S rRNA gene sequences, showing the relationships between strain KUDC0405^T^ and the related type species Bayesian posterior probability values ≥ 0.5 were shown at the nodes. Bar, 0.03 The scale bar indicates the number of substitutions per site.

The 16S rRNA gene sequence of the strain KUDC0405^T^ (1,490 bp) was determined as previously described [14]. This strain exhibited the highest 16S rRNA gene sequence similarity (97.72%) with *M. bovistercoris* NEAU-LLE^T^, followed by *M. pseudoresistence* CC-5209^T^ (97.58%), *M. resistens* NBRC 103078^T^ (97.51%), *M. oleivorans* NBRC 103075^T^ (97.51%), *M. testaceum* NBRC 12675^T^ (97.51%), and *M. paraoxydans* NBRC 103076^T^ (97.30%). A comparison of the preliminary 16S rRNA gene sequences revealed that strain KUDC0405^T^ is related to members of the genus *Microbacterium*. In the Bayesian inference tree (Fig. 1), neighbor-joining phylogenetic tree (Fig. S1), maximum likelihood tree (Fig. S2), and maximum parsimony tree (Fig. S3), strain KUDC0405^T^ was grouped with *M. bovistercoris* NEAU-LLE^T^ and *M. pseudoresistence* CC-5209^T^. KUDC0405^T^ exhibited a distinct phylogenetic lineage, indicating that it is a novel species belonging to the genus *Microbacterium* (Figs. 1, S1, S2, and S3). The GenBank/EMBL/DDBJ accession numbers for the partial 16S rRNA gene sequence of KUDC0405^T^ are MT071892 and CP091139.

### Genomic Analysis

The complete genomes determined have been deposited in the NCBI GenBank database under accession number GCF_021582895 (KUDC0405^T^). The complete genome of strain KUDC0405^T^ consisted of a circular chromosome (3,610,832 bp). The genomic DNA G+C content was 70.4%, which is within the range reported for the *Microbacterium* genus. A total of 3,654 genes were identified, of which 3,018 were protein-coding genes and 52 were RNA genes (3 rRNA, 46 tRNA, and 3 ncRNA genes). Table 1 describes the genomic features of the strain KUDC0405^T^ and closely related strains. The genome of strain KUDC0405^T^ displayed 256 subsystems according to genome annotation using RAST. Various metabolic genes were predicted for various metabolic processes, such as the metabolism of amino acids and derivatives (311 genes), carbohydrates (247 genes), cofactors, vitamins, prosthetic groups, pigments (153 genes), proteins (151 genes), nucleosides and nucleotides (106 genes), DNA (60 genes), virulence, disease, and defense (38 genes), membrane transport (33 genes), respiration (33 genes), and other metabolic processes. KUDC0405^T^ did not appear to be motile and the genome contained no genes encoding proteins associated with motility. The OrthoVenn diagram revealed orthologous protein clusters of strain KUDC0405^T^, *M. bovistercoris* NEAU-LLE^T^, and *M. pseudoresistens* CC-5209^T^ (Fig. S4). Strain KUDC0405^T^ formed 4,260 proteins, 2,019 orthologous clusters, and 2,106 singletons. All three strains (strain KUDC0405^T^, *M. bovistercoris* NEAU-LLE^T^, and *M. pseudoresistence* CC-5209^T^) shared 1,510 orthologous protein clusters (Fig. S4, Table S1). AntiSMASH 6.0 revealed that KUDC0405^T^ has the following five putative BGCs responsible for the biosynthesis of secondary metabolites: ectonine (located from 20,987 to 31,382 nt), T3PKS1 (located from 1,168,528 to 1,207,332 nt), T3PKS2, RRE-containing (located from 2,647,239 to 2,707,971 nt), and terpene (located from 3,487,598 to 3,508,218 nt) (Table S2). The terpene cluster exhibited high similarity (>80%) to the reported carotenoid gene cluster. The KUDC0405^T^ genome was compared with those of *M. bovistercoris* NEAU-LLE^T^ and *M. pseudoresistence* CC-5209^T^. All values were notably lower than their thresholds (ANI, ~95%; AAI, ~95%; dDDH, ~70%). In the case of ANIm, <20% of the genome was aligned for *M. bovistercoris* NEAU-LLE^T^, and the alignment was assigned as suspicious by the software. In silico, AAI values were analysed at 64.7% and 65.0% in strain KUDC0405^T^, *M. bovistercoris* NEAU-LLE^T^, and strain KUDC0405^T^ and *M. pseudoresistence* CC-5209^T^, respectively. Also, GGDC results for strains KUDC0405^T^, *M. bovistercoris* NEAU-LLE^T^, and *M. pseudoresistence* CC-5209^T^ were calculated as 17.3% and 17.5% based on formula 2 (identities/HSP length). The ANIb, ANIm, AAI, and dDDH values of strain KUDC0405^T^ compared with those of the closely related strains are presented in Table 2. These genome data clearly indicate that strain KUDC0405^T^ represents a novel species of the *Microbacterium* genus.

### Phenotypic, Physiological, and Biochemical Characteristics

**Fig. 2:**
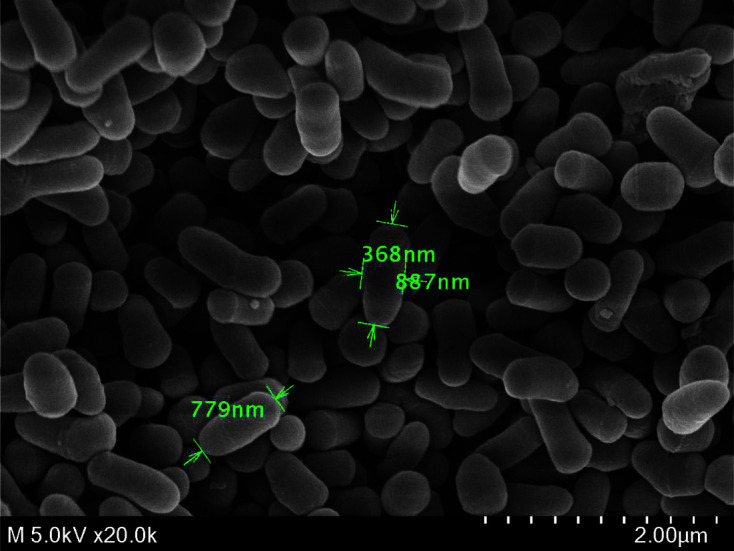
Scanning electron micrograph of KUDC0405^T^. The scanning electron micrograph of cells from strain KUDC0405^T^ grown on tryptic soy agar at 30°C for 48 h. Scale bar, 2 μm.

The strain KUDC0405^T^ was gram-positive, non-spore forming, non-motile, and grew anaerobically on TSA. Colonies on TSA media were smooth, circular, yellowish-white, and the cells were rod-shaped (0.3–0.4 × 0.7–0.8 μm) (Fig. 2). Growth was observed at 25–37°C (optimum, 25–30°C), pH 6–8 (optimum, pH 7), and with 0–7.0% NaCl (w/v) (optimum, 0.5–1.2%) on TSA under aerobic conditions. KUDC0405^T^ produces siderophores, which are iron-chelating compounds secreted by microorganisms that transport iron to the cell membranes of plants [41, 42]. The cells were oxidase-positive and catalase-positive. API ZYM strips are positive for acid phosphatase, alkaline phosphatase, crystalline arylamidase, esterase, esterase lipase, leucine arylamidase, lipase, N-acetyl-β-glucosamidase, naphthol-AS-BI-phosphohydrolase, trypsin, valine arylamidase, α-glucosidase, α-mannosidase, β-glucouronidase, and β-glucosidase, but negative for α-chymotrypsin, α-fructosidase, α-galactosidase, and β-galactosidase. Acid is produced from (API 50CH) 5-ketogluconate, aesculin, arbutin, cellobiose, D-arabinose, D-lyxose, D-tagatose, D-turanose, erythritol, fructose, galactose, glucose, L-arabinose, L-xylose, maltose, mannitol, mannose, melezitose, rhamnose, ribose, salicin, sorbose, starch, sucrose, and trehalose but not from 2-ketogluconate, adonitol, amygdalin, D-arabitol, D-fructose, dulcitol, gentiobiose, gluconate, glycerol, glycogen, inositol, inulin, L-arabitol, L-fructose, lactose, melibiose, methyl-α-D-glucoside, methyl-α-D-mannoside, methyl-β-D-xyloside, N-acetylglucosamine, raffinose, sorbitol, and xylitol. API 20NE strips were positive for glucose fermentation/oxidation, esculin hydrolysis, and nitrate reduction but negative for indole production, arginine dihydrolase, urease, gelatine hydrolysis, and adipic acid, capric acid, and trisodium citrate assimilation. Strain KUDC0405^T^ indicated hydrolysis of Tween 20, 40, and 80 but not Tween 60, starch, and urea (Table 3, Table S3).

### Chemotaxonomic Characteristics

The predominant menaquinone in KUDC0405^T^ was MK-12. The polar lipids included diphospharidylglycerol, glycolipid, phosphatidylglycerol, an unidentified phospholipid, three unidentified aminolipids, and an unidentified lipid (Fig. S5). Diphosphathydylglycerol, glycolipid, and phosphatidylglycerol were commonly identified in KUDC0405^T^, *M. bovistercoris* NEAU-LLE^T^, and *M. pseudoresistence* CC-5209^T^ (Fig. S5). Strain KUDC0405^T^ contained glycine (32.4%), ornithine (26.8%), alanine (25.4%), and glutamic (15.4%) as cell-wall peptidoglycans. The major fatty acids in KUDC0405^T^ were anteiso-C_17:0_ (35.2%), iso-C_16:0_ (16.3%), and iso-C_17:0_ (8.0%), and the minor components included iso-C_15:0_ (4.0%), C_16:0_ (2.6%), anteiso-C_15:0_ (2.5%), and C_18:0_ (1.5%). Table 4 shows the cellular fatty acid profiles of KUDC0405^T^ and the most closely related reference strains. *M. bovistercoris* NEAU-LLE^T^ and *M. pseudoresistence* CC-5209^T^ presented anteiso-C_17:0_ and anteiso-C_15:0_ as major fatty acids. The major fatty acids in the genus *Microbacterium* were anteiso-C_17:0_.

To conclude, we suggest that strain KUDC0405^T^ represents a novel species of the genus *Microbacterium*, for which we suggest the name *M. elymi* sp. nov..

### Description of *Microbacterium elymi* sp. nov. KUDC0405^T^ *M. elymi* (e’ly.mi. N. L. gen. n. elymi of the plant *E. tsukushiensis*)

Cells are Gram-stain-positive, catalase- and oxidase- positive, non-spore forming, non-motile, facultatively anaerobic and rod-shaped (0.3–0.4 × 0.7–0.8 μm). Colonies are smooth, circular, yellowish-white, and 3.0–0.4 mm in diameter on TSA with growth for 2 days. Optimal growth occurs at 25–30°C, at pH 7, and 0.5–1.2%NaCl (w/v) on TSA media. Strain KUDC0405^T^ produces siderophores and contains glycine, ornithine, alanine, and glutamic acid as cell-wall peptidoglycans. The polar lipids were diphosphatydilglycerol, glycolipid, phosphatydilglycerol, and phospholipid; the major menaquinone was MK-12; and the major fatty acids were anteiso-C_17:0_ and iso-C_16:0_. The genomic DNA GC content was 70.4%.

The type strain, KUDC0405^T^ (=KCTC 49411^T^ =CGMCC1.18472^T^), was isolated from the rhizosphere of *E. tsukushiensis* collected from the Dokdo Islands, Republic of Korea. The GenBank/EMBL/DDBJ accession numbers for the partial 16S rRNA gene sequence and genome sequence of KUDC0405^T^ were MT071892 and CP091139, respectively. NCBI accession number for genomes are GCF_021582895.

General features of the genome *de novo* assembly are as follows: genome size, 3,610,832 bp; number of contigs, 1; coverage, 119.0 ×.

## Supplemental Materials

Supplementary data for this paper are available on-line only at http://jmb.or.kr.

## Figures and Tables

**Table 1:** Genome features of strain KUDC0405^T^ (ASM2158289), *Microbacterium bovistercoris* NEAU-LLE^T^ (ASM338757) and *M. pseudoresistens* CC-5209^T^ (ASM1340974).

Attribute	KUDC0405^T^	NEAU-LLE^T*^	CC-5209^T*^
Sequencing coverage	119	500	1376
Genome size (bp)	3610832	3743511	2914491
DNA G+C content (%)	70.4	69.3	68.9
Number of Contigs (with PEGs)	1	48	1
Genes (total)	3654	3557	2859
CDCs (total)	3596	3496	2806
CDCs (with protein)	3018	3426	2673
rRNAs	3	3	3
tRNAs	46	48	44
ncRNAs	3	3	3

*Data obtained by Ling *et al*. (2019) and Young *et al*. (2010).

**Table 2:** The ANIb, ANIm, AAI, tetra-z score and GGDC analysis of strain KUDC0405^T^ and related type strains.

Strain	ANIb (%)	ANIm (%)	AAI (%)	Tetra-z score	GGDC (%)
NEAU-LLE^T^	74.29	84.13	64.66	0.95856	17.3
CC-5209^T^	74.36	84.53	64.97	0.89333	17.5

The ANI, AAI, tetra-z core and GGDC values between strain KUDC0405^T^ and reference strains (*Microbacterium bovistercoris* NEAU-LLE^T^ and *M. pseudoresistens* CC-5209^T^).

**Table 3:** The characteristics analysis of strain KUDC0405^T^ and the type strains of closely related species of the genus Microbacterium*.

Characteristic	1	2	3
Isolation source	Rhizosphere	Cow dung	Stalk of the edible mushroom
Colony color	Yellowish-white	Light yellow	Yellow
Optimal growth			
Temperature (°C)	25–30	25–30	25–30
pH	7	7	7
Catalase	+	+	+
Oxidase	+	+	+
Hydrolysis of Starch	-	-	-
API 20NE:			
Reduction of nitrates to nitrites, NO_3_ → NO_2_	+	-	-
Reduction of nitrates to nitrogen, NO_3_ → N_2_	-	-	-
Indole production	-	-	-

All strain are Gram-positive and rop-shaped cells. In addition, all species form endospore and grow aerobically. Strains; 1, KUDC0405^T^; 2, *Microbacterium bovistercoris* NEAU-LLE^T^; 3, *M. pseudoresistens* CC-5209^T^.

**Table 4:** Cellular fatty acid profiles of strain KUDC0405^T^ and the closely related type strains of Microbacterium* species.

Fatty acid (%)	1	2	3
Saturated:			
C_16:0_	2.6	1.9	1.0
Methyl-branched:			
iso-C_15:0_	4.0	9.4	2.1
anteiso-C_15:0_	2.5	39.1	29.3
iso-C_16:0_	16.3	20.4	9.2
iso-C_17:0_	8.0	3.5	1.7
anteiso-C_17:0_	35.2	23.5	56.2

Strains; 1, KUDC0405^T^; 2, *Microbacterium bovistercoris* NEAU-LLE^T^; 3, *M. pseudoresistens* CC-5209^T^.
