# Supplementary material for: Microbacterium elymi sp. nov., Isolated from the Rhizospheric Soil of Elymus tsukushiensis, a Plant Native to the Dokdo Islands, Republic of Korea
Source: J Microbiol Biotechnol. 2023 Jan 6;33(2):188–94. doi: 10.4014/jmb.2211.11024 (PMC9998209; doi:10.4014/jmb.2211.11024)
Supplement: Supplementary file 1 [file jmb-33-2-188-supple.pdf]

J. Microbiol. Biotechnol.
https://doi.org/10.4014/jmb.2211.11024

Supplementary Figures and Tables

Figure 1. Neighbor-joining phylogenetic tree, reconstructed by comparative analysis of 16S rRNA gene sequences, showing the relationships between strain KUDC0405^{T} and the related type species. Numbers at the nodes indicate levels of bootstrap support (%) based on 1,000 resampled datasets (showing bootstrap values above ≥50%). Solid circles indicate that the corresponding nodes were obtained in both maximum-parsimony and maximum-likelihood trees. Open circles indicate that the corresponding nodes were obtained in maximum-parsimony and maximum-likelihood trees. Bar, 0.001 nucleotide substitutions per positions.

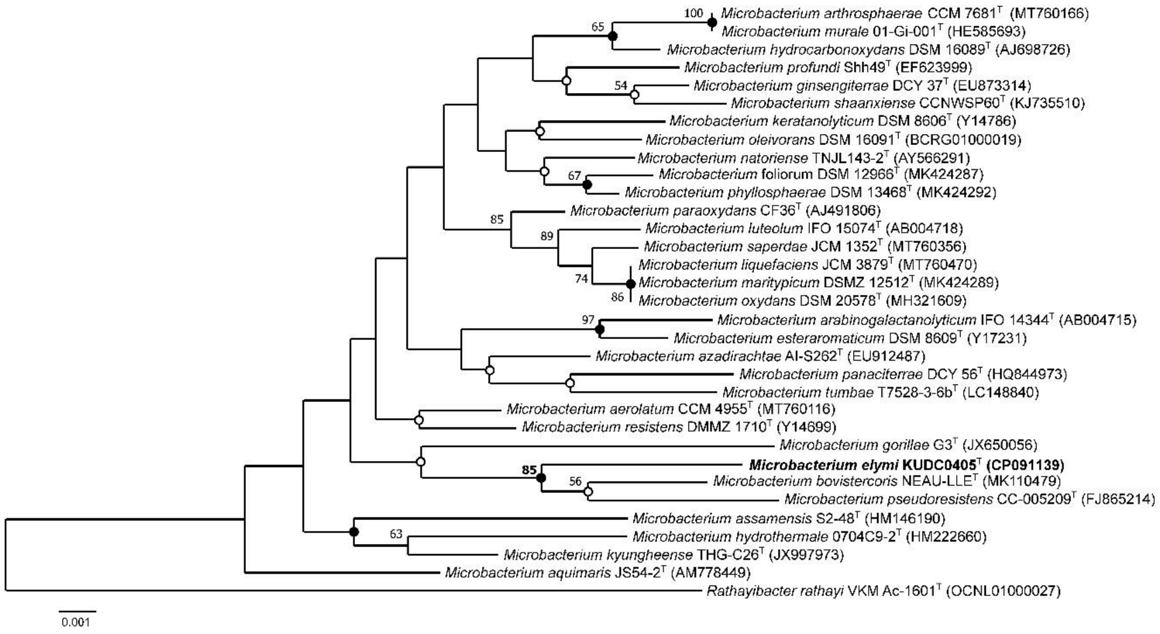

Fig. S2. The maximum-likelihood (ML) phylogenetic tree was constructed using 16S rDNA sequence of strain KUDC0405^T and its related type strains. The numbers suggest bootstrap support levels (%) based on 1000 replicated datasets. The *Rathayibacter rathayi* VKM Ac-1601^T was used as an outgroup.

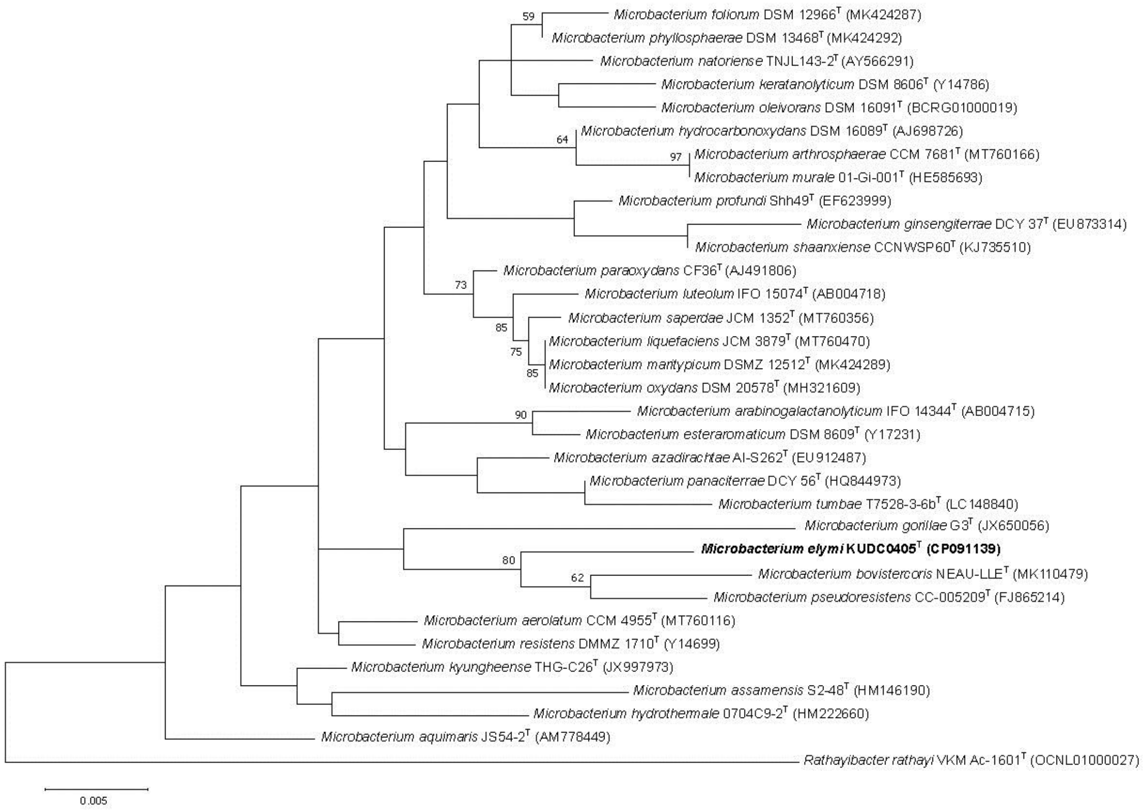

Fig. S3. The maximum-parsimony (MP) tree of KUDC0405^T and its related type strains based on 16S rRNA gene sequences. The numbers suggest bootstrap support levels (%) based on 1000 replicated datasets. The *Rathayibacter rathayi* VKM Ac-1601^T was used as an outgroup.

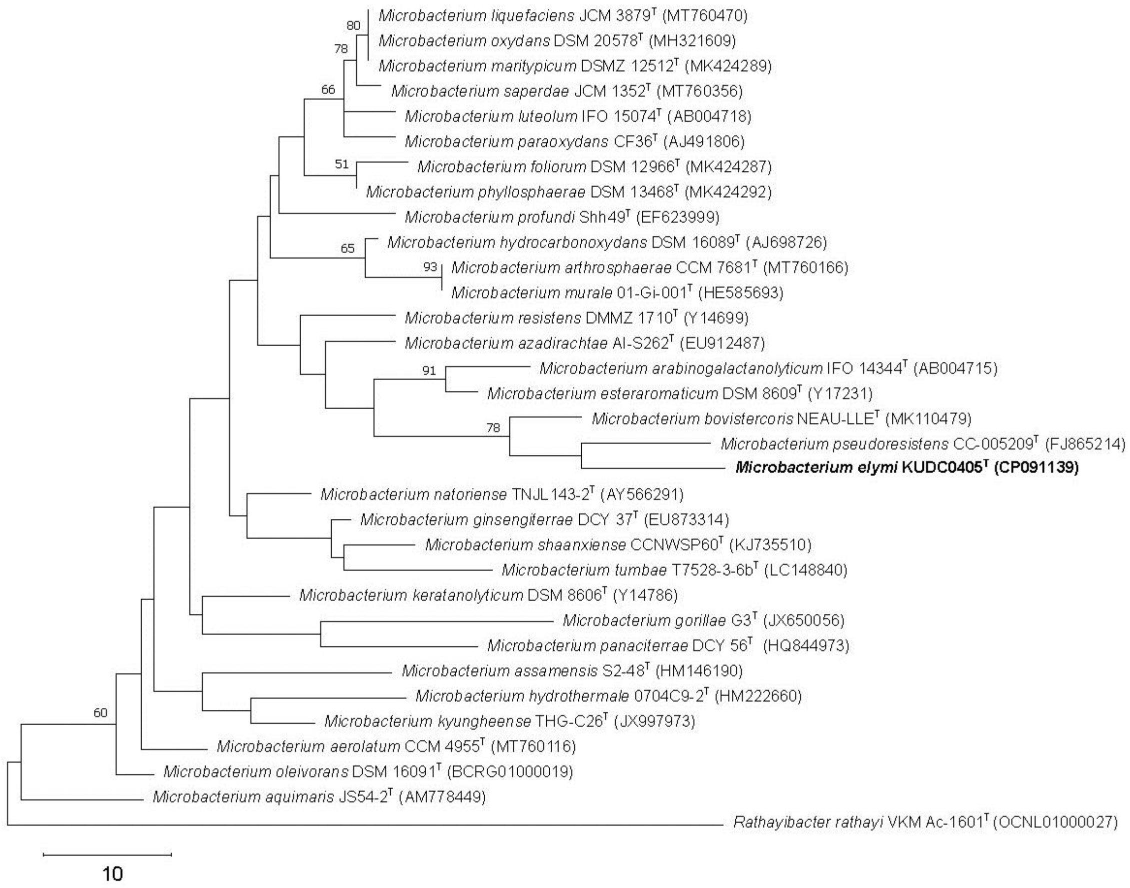

Fig. S4. Venn diagram of orthologous gene clusters among between KUDC0405^{T}, *Microbacterium bovistercoris* NEAU-LLE^{T} and *M. pseudoresistence* CC-5209^{T}. MB, *Microbacterium bovistercoris* NEAU-LLE^{T}; MP, *M. pseudoresistence* CC-5209^{T}.

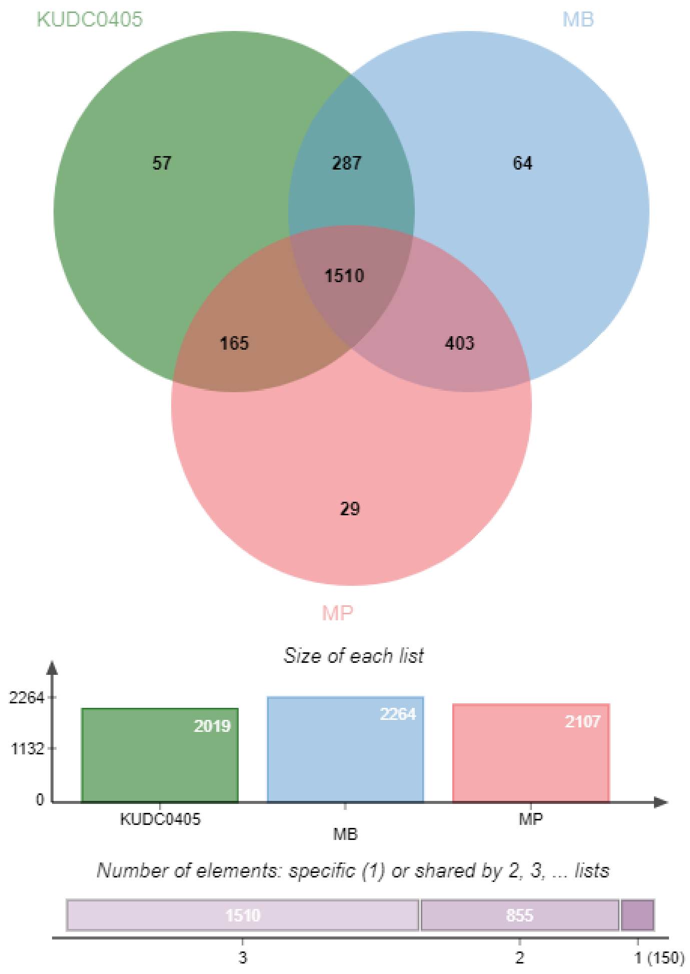

Fig. S5. Polar lipids profile of KUDC0405^T and related type strains. The polar lipids were identified with reagents 5% w/v ethanol molybdophosphoric acid from (a) strain KUDC0405^T, (b) *Microbacterium bovistercoris* NEAU-LLE^T and (c) *M. pseudoresistens* CC-5209^T. DPG, diphosphatidylglycerol; GL, glycolipid; PG, phosphatidylglycerol; PL, phospholipid; AL, unidentified aminolipid; UL, unidentified lipid

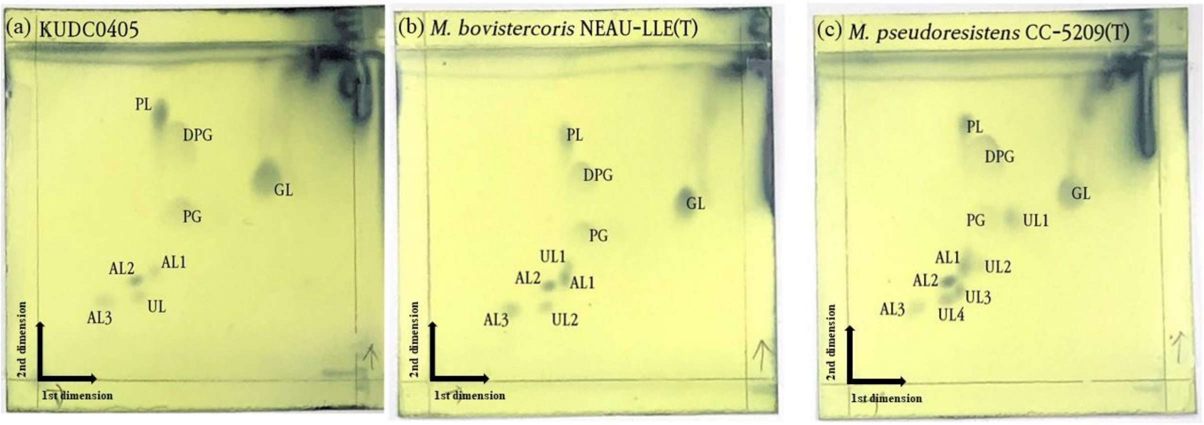

Table S1. The orthologous Venn diagram summary of KUDC0405^T and related reference type strains.

Strain	Total proteins	Orthologous clusters	Singletons
KUDC0405^T	4260	2019	2106
M. bovistercoris NEAU-LLE^T	3426	2264	1039
M. pseudoresistens CC-5209^T	2817	2107	629

Table S2. The BGCs (biosynthetic gene clusters) of KUDC0405^T and closet type strains responsible for the biosynthesis of secondary metabolites were revealed by AntiSMASH 6.0.

Strain	Cluster type	Most similar known cluster	location
KUDC0405^T	ectoine	ectoine	from 20,987 to 31,382
	T3PKS	-	from 1,168,528 to 1,207,332
	T3PKS, RRE-containing	merochlorin A / merochlorin B / deschloro-merochlorin A/ deschloro-merochlorin B / isochloro-merochlorin B/ dichloro-merochlorin B / merochlorin D / merochlorin C	from 2,647,239 to 2,707,971
	terpene	carotenoid	from 3,487,598 to 3,508,218
M. bovistercoris NEAU-LLE^T	furan, RiPP-like	-	from 1 to 14,970
	terpene	carotenoid	from 108,582 to 129,451
	betalactone	microansamycin	from 242,259 to 270,448
M. pseudoresistens CC-5209^T	ectoine	ectoine	from 1,124,036 to 1,134,428
	RRE-containing	-	from 1,633,234 to 1,654,598
	betalactone	microansamycin	from 2,821,710 to 2,849,204

Table S3. Chemotaxonomic tests of KUDC0405^{T} and related reference type strains. Strains; 1, KUDC1714^{T}; 2, *Microbacterium bovistercoris* NEAU-LLE^{T}; 3, *M. pseudoresistens* CC-5209^{T}.

Characteristic	1	2	3
**Enzymic activities**			
D-cellobiose	+	+	-
D-fucose	-	-	-
D-lactose	-	+	-
D-xylose	+	+	+
Erythritol	+	-	+
L-arabitol	-	-	-
Methyl-α D-glucoside	-	+	+
Methyl-β D-xyloside	-	+	-
N-acetylglucosamine	-	-	+
Potassium 2-ketoglyconate	-	+	-
Potassium 5-ketogluconate	+	+	+
Xylitol	-	-	+
**Carbon source utilization :**			
D-arabinose	+	+	-
L-arabinose	+	+	-
D-fructose	+	+	+
D-galactose	+	+	+
D-glucose	+	+	-
Glycerol	-	-	-
D-maltose	+	+	-
D-mannitol	+	-	+
D-mannose	+	+	-
D-melibiose	-	-	-
D-raffinose	-	+	-
L-xylose	+	-	+
Salicin	+	+	-
